# Seeing Is Believing: Neural Representations of Visual Stimuli in Human Auditory Cortex Correlate with Illusory Auditory Perceptions

**DOI:** 10.1371/journal.pone.0073148

**Published:** 2013-09-04

**Authors:** Elliot Smith, Scott Duede, Sara Hanrahan, Tyler Davis, Paul House, Bradley Greger

**Affiliations:** 1 Interdepartmental Program in Neuroscience, University of Utah, Salt Lake City, Utah, United States of America; 2 Department of Bioengineering, University of Utah, Salt Lake City, Utah, United States of America; 3 Department of Linguistics, University of Utah, Salt Lake City, Utah, United States of America; 4 Department of Neurosurgery, University of Utah, Salt Lake City, Utah, United States of America; University of Southern California, United States of America

## Abstract

In interpersonal communication, the listener can often see as well as hear the speaker. Visual stimuli can subtly change a listener’s auditory perception, as in the McGurk illusion, in which perception of a phoneme’s auditory identity is changed by a concurrent video of a mouth articulating a different phoneme. Studies have yet to link visual influences on the neural representation of language with subjective language perception. Here we show that vision influences the electrophysiological representation of phonemes in human auditory cortex prior to the presentation of the auditory stimulus. We used the McGurk effect to dissociate the subjective perception of phonemes from the auditory stimuli. With this paradigm we demonstrate that neural representations in auditory cortex are more closely correlated with the visual stimuli of mouth articulation, which drive the illusory subjective auditory perception, than the actual auditory stimuli. Additionally, information about visual and auditory stimuli transfer in the caudal–rostral direction along the superior temporal gyrus during phoneme perception as would be expected of visual information flowing from the occipital cortex into the ventral auditory processing stream. These results show that visual stimuli influence the neural representation in auditory cortex early in sensory processing and may override the subjective auditory perceptions normally generated by auditory stimuli. These findings depict a marked influence of vision on the neural processing of audition in tertiary auditory cortex and suggest a mechanistic underpinning for the McGurk effect.

## Introduction

The McGurk effect is an auditory illusion that occurs when the perception of a phoneme’s auditory identity is changed by a concurrently played video of a mouth articulating a different phoneme [1]. Most subjects will report hearing the phoneme articulated by the mouth in the video and not the different phoneme pronounced in the auditory stimulus [2]. The concurrent visual stimulus is presumably altering the neural representation, and therefore subjective perception, of the auditory stimulus. Understanding how, and where, neural representations are changed and perceptual identity is altered will provide important insight into the neural mechanisms of everyday speech perception.

The perceptual identity of a sound is thought to be processed hierarchically in the human brain along the superior temporal lobe in a cortical processing stream analogous to the ventral visual processing stream in the inferior temporal lobe [3], [4], [5], [6]. Studies of the neural representation of language have therefore focused on the neural construction of phonemic identity in the superior temporal lobe. Electrical recordings from the surface of the human brain have determined that local field potentials correlate with subjective phoneme categorization [7] and show topographic coding of specific speech sounds in the superior temporal gyrus (STG) [8], [9].

Where in the brain, and to what extent, vision influences auditory perception is not well understood. Visual enhancement and suppression of auditory responses have been observed at the level of primary auditory cortex (AI) in macaques [10], [11]. Electrophysiological recordings through the medial to lateral extent of the human temporal lobe have determined that vision influences audition early on during a neural response and that visual influence extends to hierarchically lower cortical areas [12], [13], [14]. Magnetoencephalography and electroencephalography showed that auditory representations in the superior temporal lobe were altered by visual influences, an idea that has cultivated the argument that visual influences play a predictive role in determining speech identity [15], [16]. These visual influences on auditory cortex, however, have not been linked to subjective perception. Understanding how visual influences alter auditory perception during the McGurk illusion, a potent example of vision’s effect on auditory perception, will provide insight into the neural mechanisms of quotidian speech perception.

To explore this issue, we examined electrical activity recorded from subdural electrodes in four human patients (two right hemispheres and three left hemispheres; we recorded from one patient bilaterally [see materials and methods]) with pharmacologically intractable epilepsy who were undergoing monitoring for seizure activity. The analyses herein are based on broadband electrical potentials recorded from the surface of the human auditory cortex. These electrical signals are believed to represent “both action potentials and other membrane potential-derived [electrical] fluctuations in a small neuronal volume” [17] surrounding the electrode; they have been shown to be related to action potentials and provide valuable information about neural coding [18], [19]. Using the McGurk effect, we were able to dissociate the identity of an auditory perception from the auditory stimulus provided to the ear. Here we show that neural representations of the McGurk effect in human parabelt auditory cortex correlate with illusory subjective perception of the stimulus more than with the actual auditory stimulus.

## Results

**Figure 1:**
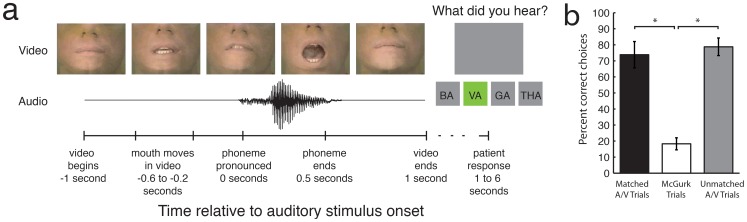
Task description and performance. **a,** The task consisted of a video of a mouth pronouncing one of four phonemes. This video was randomly paired with audio of a male pronouncing one of the same four syllables. The video times here are shown in the text below the timeline. There was one second of video before the audio began, during which the mouth moved slightly in order to position to speak the starting phoneme. The audio syllable lasted half a second, and there was one second of video after the audio had finished. After a brief randomized delay, the subject was cued to respond. The patient had five seconds to respond before a new trial was initiated. **b,** Task performance for three conditions. Patients performed significantly better on Matched A/V (73.81%, N = 186) trials and Unmatched A/V (78.77%, N = 299) trials when compared with McGurk (18.29%, N = 152) trials (ANOVA, Tukey-Kramer method for multiple comparisons, p<0.01 for both comparisons).

Subjects performed an audiovisual speech perception task in which a video stimulus of a mouth articulating one of four phonemes (“BA,” “GA,” “VA,” and “THA”) was paired with an audio stimulus of one of the same four phonemes (/BA/,/GA/,/VA/, and/THA/) (Fig. 1a). Video and audio stimuli were randomly paired, creating 16 possible stimulus combinations. After each audiovisual stimulus had been delivered, four buttons in the task control software appeared, cueing the subject to indicate which phoneme he or she had heard. We grouped these trials into three categories. The first category, “Matched A/V” trials (N = 186), were those trials in which the audio and video stimuli had the same phonemic identity. Trials in which the audio and video stimuli did not match were grouped into two categories: “McGurk” trials (N = 152) were those in which the video and audio stimuli did not match and the patient reported hearing the phonemic identity of the video stimulus and not the phonemic identity of the audio stimulus, and “Unmatched A/V” trials (N = 299) were those in which the video and audio stimuli did not match and did not produce a McGurk illusion. Patients performed significantly better at identifying the audio stimuli on Matched A/V (73.81%) trials and Unmatched A/V (78.77%) trials compared with McGurk (18.29%) trials (ANOVA, Tukey-Kramer method for multiple comparisons, p<0.01) (Fig. 1b).

**Figure 2:**
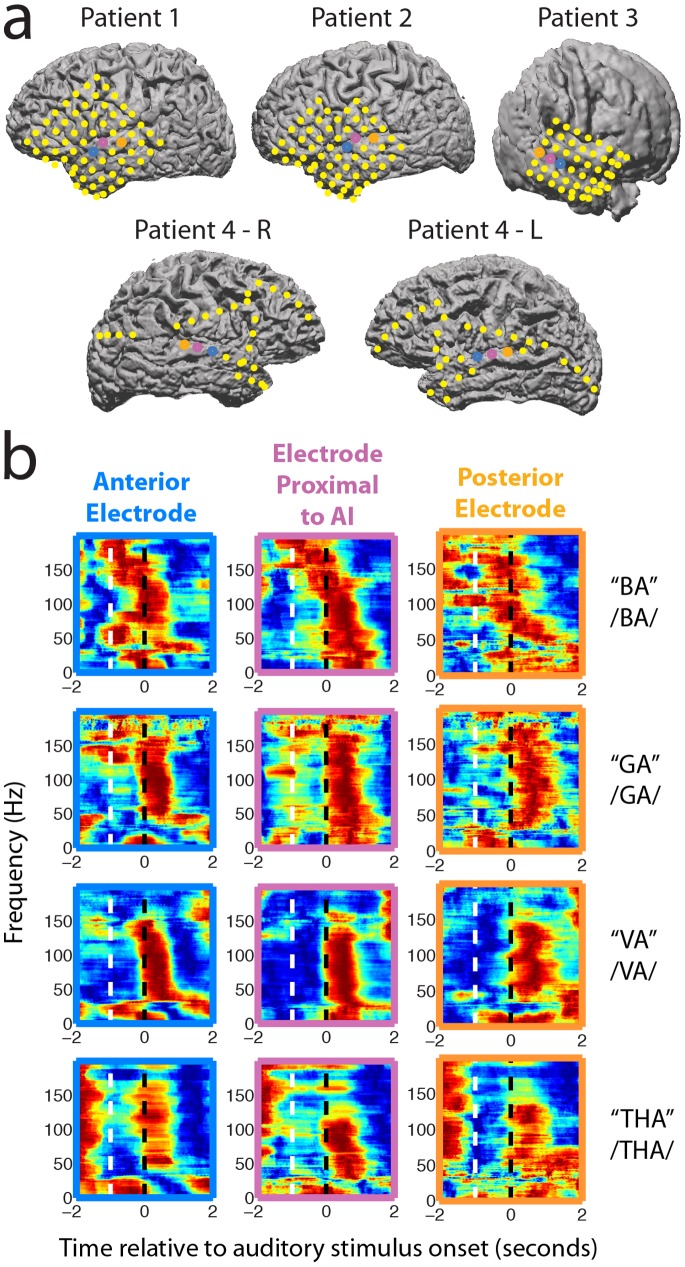
Electrode locations and responses to auditory stimuli on STG electrodes. **a,** Electrode locations for five hemispheres in four patients. Yellow dots indicate electrode placements we did not use for analyses. Electrodes used for analysis have been color coded. Blue represents the anterior electrode, pink represents the electrode proximal to AI, and orange represents the posterior electrode. **b,** Representative neural responses for each phoneme on three STG electrodes. White dashed lines indicate the start of the video. Black dashed lines indicate the start of the audio. Responses are outlined to match their respective electrode locations.

We restricted our analyses of neural signals to three electrodes per patient. These were the electrodes with the greatest spectral power in the 75–200 Hz range during the 1000 ms when the auditory phoneme is pronounced. Further analysis of the spatial location of these electrodes, based on preoperative magnetic resonance (MR) images and postoperative computed tomography (CT) images [20], indicated that all three electrodes were on STG in Brodmann’s areas 41 and 42, or parabelt auditory cortex (Fig. 2a). Figure 2b shows example responses averaged over Matched A/V trials for one patient. For all patients, we observed bursts in spectral power coincident with the presentation of auditory stimuli on all three of the electrodes that were used for analysis.

**Figure 3:**
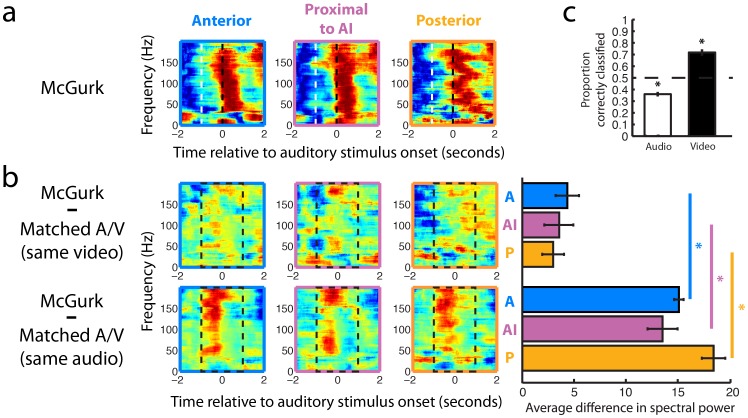
Visual representations in parabelt auditory cortex. **a,** Example spectrograms for the McGurk condition (“VA” &/BA/). Spectrograms were normalized by frequency band. White dotted lines indicate the start of the video. Black dotted lines indicate the start of the audio. **b,** Example difference spectrograms for all three electrodes from one patient. Matched A/V spectrograms were subtracted from McGurk spectrograms (“VA” &/BA/− “VA” &/VA/and “VA” &/BA/− “BA” &/BA/) between −1 and 1 seconds relative to auditory stimulus onset (between black dashed lines in spectrograms). McGurk spectrograms were significantly less different from Matched A/V spectrograms with the same video identity than Matched A/V spectrograms with the same audio identity, as shown in the bar graph to the left of the difference spectrograms. Electrode locations are color coded and labeled (A, anterior electrode; AI, electrode proximal to AI; P, posterior electrode). **c,** A statistical classifier accurately classified McGurk trials when tested on the identity of the video (74.33%); however, the classifier consistently chose the wrong auditory identity for McGurk trials (36.17%). The dashed line represents chance level classification (50.00%).

The McGurk effect is robust enough to be perceived even if the viewer knows the illusion is occurring, suggesting that visual stimuli can influence early representations of auditory stimuli. We wanted to examine how far visual influences extend into the auditory cortex and to what extent these visual influences alter neural representation in the auditory cortex. We began by examining the similarity of neural representations during McGurk and Matched A/V condition stimuli. Spectrograms from McGurk condition trials (Fig. 3a) were similar to spectrograms from Matched A/V condition when they had the same video stimuli, even though the auditory stimuli were different. Conversely, spectrograms from McGurk condition trials were dissimilar to spectrograms from Matched A/V condition when they had the different video stimuli, even though the auditory stimuli were same. This trend was evident in data recorded from all three parabelt electrodes. To quantify this observation, the mean differences between neural responses on each electrode to the McGurk condition and the Matched A/V condition were compared for all four patients. For each electrode, spectral differences between McGurk spectrograms and Matched A/V spectrograms that had the same audio stimuli but different video stimuli were significantly greater than the spectral differences between McGurk spectrograms and Matched A/V spectrograms that had different audio stimuli and the same video stimuli (Mann-Whitney U test, p<0.01 ) (Fig. 3b). This result indicates that in parabelt auditory cortex, neural representations of the McGurk illusion corresponded to the video stimuli more than the audio stimuli, i.e., the neural representation of the visual stimulus was closer to the patients’ illusory auditory perceptions.

To further probe the electrophysiological representation of audiovisual language stimuli in parabelt auditory cortex on a trial-by-trial basis, a statistical classifier was trained on local field potential waveforms and spectrograms from all three electrodes for half of the McGurk trials. This classifier was then used to decode the identity of the video or the audio stimuli of each trial from the remaining half of the McGurk trials [21], [22]. The training and testing sets were then switched to obtain a twofold cross-validation. Classifications were conducted for each pairwise comparison, i.e., chance performance was 50%. Classifier performance was significantly better than chance for McGurk stimuli when the correct classification was chosen as the video identity (Wilcoxon signed rank test, p<0.01); however, the classifier consistently chose the wrong identity when the correct classification was chosen as the sound identity (Wilcoxon signed rank test, p<0.01) (Fig. 3c). Pairwise classification of both audio and video identity was also significantly different from chance within subjects, over each pairwise classification (Wilcoxon signed-rank test, N = 16, p<0.001). These results indicate that trial-by-trial neural representations for phoneme stimuli in parabelt auditory cortex encoded the identity of the video stimuli during McGurk condition trials.

Visual information about object identity likely flows from caudal to rostral into the auditory cortex along the ventral visual pathway [6] and similarly flows from caudal to rostral onto frontal cortex along the ventral auditory pathway [4], [5]. To examine this idea, we used a conditional information theoretic analysis [23], [24] to determine the transfer of information about visual or auditory stimulus identity among the three STG electrodes (Fig. S1). This analysis quantifies the average reduction in uncertainty about the identity of a stimulus from observing a neural response, given that we already know the response identity in another area. Information transfer was examined for three, one-second time intervals: 1) a baseline interval in which no audio or video stimulus is present; 2) the interval when the video of the mouth articulation is being shown, yet the audio stimulus has not begun; and 3) the interval when the audio stimulus of the phoneme being pronounced and the video stimulus of the mouth articulation are being concurrently presented.

**Figure 4:**
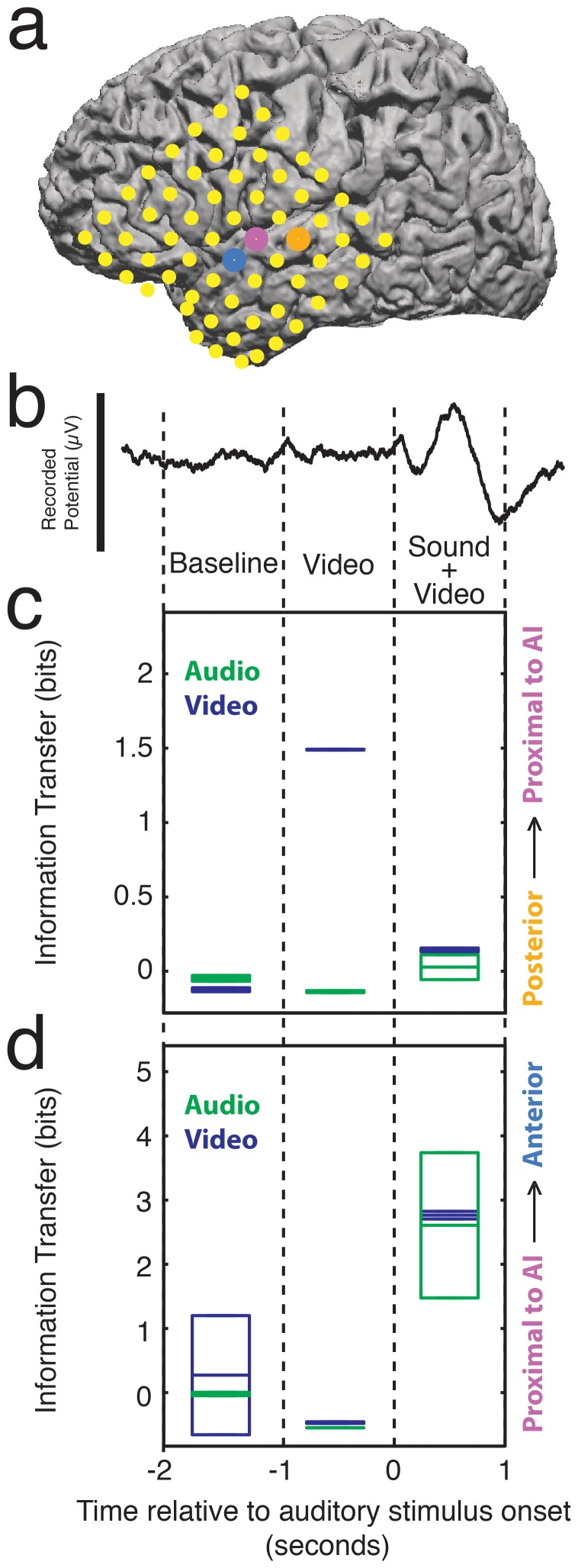
Transfer information in the superior temporal lobe. **a,** Electrode locations from Patient 1 for electrode location and information transfer directionality reference. **b,** An averaged evoked potential from the middle electrode is shown above the plots for reference. The scale bar for the evoked potential is 400 µV. **c,** Plot of information transfer between the posterior electrode and the electrode proximal to AI for 3-second time periods through the duration of the trial. **d,** Plot of information transfer between the electrode proximal to AI and the anterior electrode for 3-second time periods through the duration of the trial. For both **b** and **c**, positive values indicate transfer of information in the caudal–rostral direction, and negative values indicate transfer of information in the rostral–caudal direction. Green box plots indicate information about the identity of the audio stimuli. Blue plots indicate information about the video stimuli. Box plots show means and quartiles for the 5 hemispheres.

Information transfer about the identity of the visual stimuli was increased between the posterior electrode and the electrode proximal to AI during the period when the mouth begins articulating the phoneme, yet no auditory stimulus is present (Fig. 4a, 4b). Information about the phonemic identity from the video stimuli was transferred in the caudal–rostral direction along the STG before the onset of the auditory stimuli, suggesting that the ventral visual “what” pathway is providing input into cortical areas in the superior temporal lobe early on during audiovisual language perception. Information transfer about the identity of both the visual and auditory stimuli was increased between the electrode proximal to AI and the anterior electrode during the period when the visual stimulus of mouth articulation and the auditory stimulus of phoneme pronunciation were concurrently presented (Fig. 4a, 4c). The temporal dynamics of the caudal-to-rostral information transfer along the STG show early visual information influencing the neural representation of phonemes in auditory cortex and later visual and auditory information passing into the audition-for-perception processing stream [5].

## Discussion

Results from electrocorticography in human STG depict speech representations in tertiary auditory cortex as being altered by attention or the context of a sound [25] and suggest multimodal influences on the early stages of auditory processing [26]. We demonstrate that auditory representations in the STG are altered by early visual stimuli and that these visual influences are predictive in determining the subjective perception of phonemes, i.e., the neural representations of phonemic identity from visual input can extend into auditory cortex and affect the perception of language. The time course and direction of auditory and visual information transfer about the identity of phonemes in parabelt auditory cortex showed that visual information transfers caudorostrally through STG before any auditory stimuli were presented. This suggests a mechanistic underpinning for the McGurk effect in which the information from the visual cortex may be instructing the auditory cortex which phoneme to “hear” before an auditory stimulus is received. This understanding of multisensory neocortical language processing provides insight into the multisensory neural mechanisms underlying quotidian language perception and has implications for rehabilitation therapy and neural prostheses.

## Materials and Methods

### Ethics Statement

These experiments were approved by the University of Utah Institutional Review Board. All patients in this study provided written consent. The consent process was approved by the University of Utah Institutional Review Board.

### Subjects

Electrocorticography (ECoG) electrodes were implanted in four human patients for clinical monitoring of epilepsy. Frontotemporal ECoG grids with 64 electrodes in the left hemisphere were used in patients 1 and 2, a frontotemporal grid with 48 electrodes in the right hemisphere was implanted in patient 3, and strips of ECoG electrodes in both the left and right hemispheres were used in patient 4 (Fig. 1a). Recordings were made from both hemispheres at the same time in this patient.

### Task Design and Behavioral Testing

The patients performed a multisensory speech perception task in which syllables were delivered binaurally from flat frequency response, closed-back headphones concurrently with videos of a mouth articulating phonemes shown on a monitor. Four audio (/BA/,/GA/,/VA/, and/THA/) and four video (“BA,” “GA,” “VA,” and “THA”) syllables were randomly paired, creating 16 stimulus combinations. Audio syllables were all from the same male speaker and were paired with commonly used McGurk stimulus videos [27].

Stimulus combinations were grouped into three categories based on the patients’ behavioral responses: “Matched A/V” trials were those in which the video and audio had the same phonemic identity; “McGurk” trials were all trials in each stimulus combination in which the video and audio had different phonemic identities and the patient reported hearing the identity of the video more often than chance; “Unmatched A/V” trials were all trials in each stimulus combination in which the video and audio had different phonemic identities and the patient reported hearing the audio identity more often than chance.

### Data Collection and Preprocessing

Neural data were collected using a Neuroport system (Blackrock Microsystems). Electrophysiological signals were pseudodifferentially amplified at a gain of 5000× and sampled at 10 kilosamples per second for patients 1, 3, and 4. Data for patient 2 were sampled at 1 kilosample per second. All data were low-pass filtered at 500 Hz and downsampled to 1 kHz for further analysis. Data for each electrode were then re-referenced against all other ECoG electrodes in the same hemisphere for each patient by subtracting the mean across electrodes for each trial. This re-referencing procedure acts as a large, low-impedance monopolar reference.

Multitapered spectral analysis was used to generate spectrograms [28]. A 500-millisecond moving window and 10-millisecond step size, with 7 and 11 leading tapers, were used to generate spectrograms for trial averaged spectral analysis. Averaged spectrograms were subtracted, and the mean of the absolute value of the resulting difference spectrogram is quantified in Fig. 3b.

### Electrode Selection

For each patient, three electrodes in the STG were used for analysis. These were the electrodes with the greatest spectral power between 75 and 200 Hz during auditory stimulus presentation, averaged over all stimuli. Electrodes were localized using custom software [20] based on Statistical Parametric Mapping 8 (functional neuroimaging group, University College London). After coregistering and reslicing anatomical preoperative MR images and postoperative CT images, electrodes from the CTs were projected onto cortical surfaces generated from the MR images. Patient 3′s MR images were taken more than a year before the CTs, and parts of his brain were removed before the CTs were taken, so the cortical rendering is rougher than that of the other three patients. The superior temporal and transverse temporal gyri were still visible in Patient 3′s rendered cortex. For all 5 hemispheres, the middle electrode was defined as the electrode closest to A1 based on the aforementioned electrode localization method. These middle electrodes were also defined physiologically as exhibiting the largest evoked potentials averaged over all auditory stimuli as observed during data preprocessing.

### Classification of Phoneme Identity

Single-trial spectrograms and voltage traces ranging from the onset of the video to the end of the phoneme were used as neural features in the statistical classifier [21], [24]. These multidimensional data were unwrapped to produce a two-dimensional matrix in which each row contained all the voltage, time, and frequency features from all channels for a single trial. The feature matrix was z-scored, orthogonalized using principal component analysis, and projected into the principal component space using a sufficient number of leading principal components to retain 95% of the variance in the data. Based on these neural features, data were then classified on a trial-by-trial basis using linear discriminant analysis [29]. The classifier was trained on half the trials and tested on the other half. Training and testing sets were then interchanged to obtain twofold cross-validation. Classification accuracy was measured against the level of chance, which was 50% for all classifications. The one-sample Wilcoxon signed-rank test was used to determine the level of significance for classification results.

### Information Transfer Measures

Conditional mutual information measures were calculated using probability distributions derived from the pairwise classification frequencies generated from all statistical classifications [24]. The equation
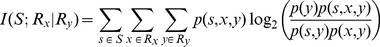
(1)was evaluated for these probability distributions, where *R_x_* and *R_y_* are the response identities on each of two electrodes, and *p(x)* and *p(y)* are the corresponding probability distributions generated from pair wise classification frequencies. We define information tendency as the net information transfer between adjacent electrodes. Information tendency is therefore the difference between the two opposing conditional mutual information measures for adjacent electrodes (e.g., *I(S;R_x_|R_y_) – I(S;R_y_|R_x_)* indicates information transfer from electrode Y to electrode X).

### Statistical Analysis

Tests for statistical significance were performed on the patients’ behavioral performance (Fig. 1b), the trial-averaged spectrogram differences (Fig. 2b), and the classification results (Fig. 2c). The patients’ behavioral analyses (Fig. 1b) were tested with an ANOVA across trial types. Pairwise comparisons of each trial type were tested with the Tukey-Kramer method for multiple comparisons. The trial-averaged spectrogram difference comparisons (Fig. 2b) were quantified by taking the mean value of the two-dimensional difference spectrogram between −1 and 1 seconds, relative to the onset of the auditory stimulus. These mean values were then tested over all patients using a Mann-Whitney U test for each electrode. The average pairwise classification performance from both runs of the twofold cross-validation over all patients (Fig. 2c) was tested using a Wilcoxon signed-rank test. Classification results were tested against a distribution with a median equal to chance performance (50%). Pairwise classification of both audio and video identity within subjects was also tested over each pairwise classification, i.e., each of 8 combinations of syllables by two cross-validations (Wilcoxon signed-rank test, N = 16, p<0.001).

## Supporting Information

Figure S1 **a,** A visual description of the statistical classifier used to classify stimulus identity. We began with spectrograms and voltage traces for each trial over all channels. All neural features were unwrapped along the trials dimension, and principal components analysis (PCA) was applied to this matrix. The PCA reconstruction determined from the training set was then applied to the testing set and linear discriminate analysis (LDA) was used to classify the identity of the syllable for each trial. **b,** Derivation of probability distributions for conditional mutual information analyses, taken from pairwise classification frequencies. A confusion matrix of pairwise classification frequencies was generated for each electrode and divided by the total number of trials to generate probability distributions for the conditional mutual information equation as shown above.
(TIF)
