# Supplementary figures and images for: Seeing Is Believing: Neural Representations of Visual Stimuli in Human Auditory Cortex Correlate with Illusory Auditory Perceptions
Source: PLoS One. 2013 Sep 4;8(9):e73148. doi: 10.1371/journal.pone.0073148 (PMC3762867; doi:10.1371/journal.pone.0073148)

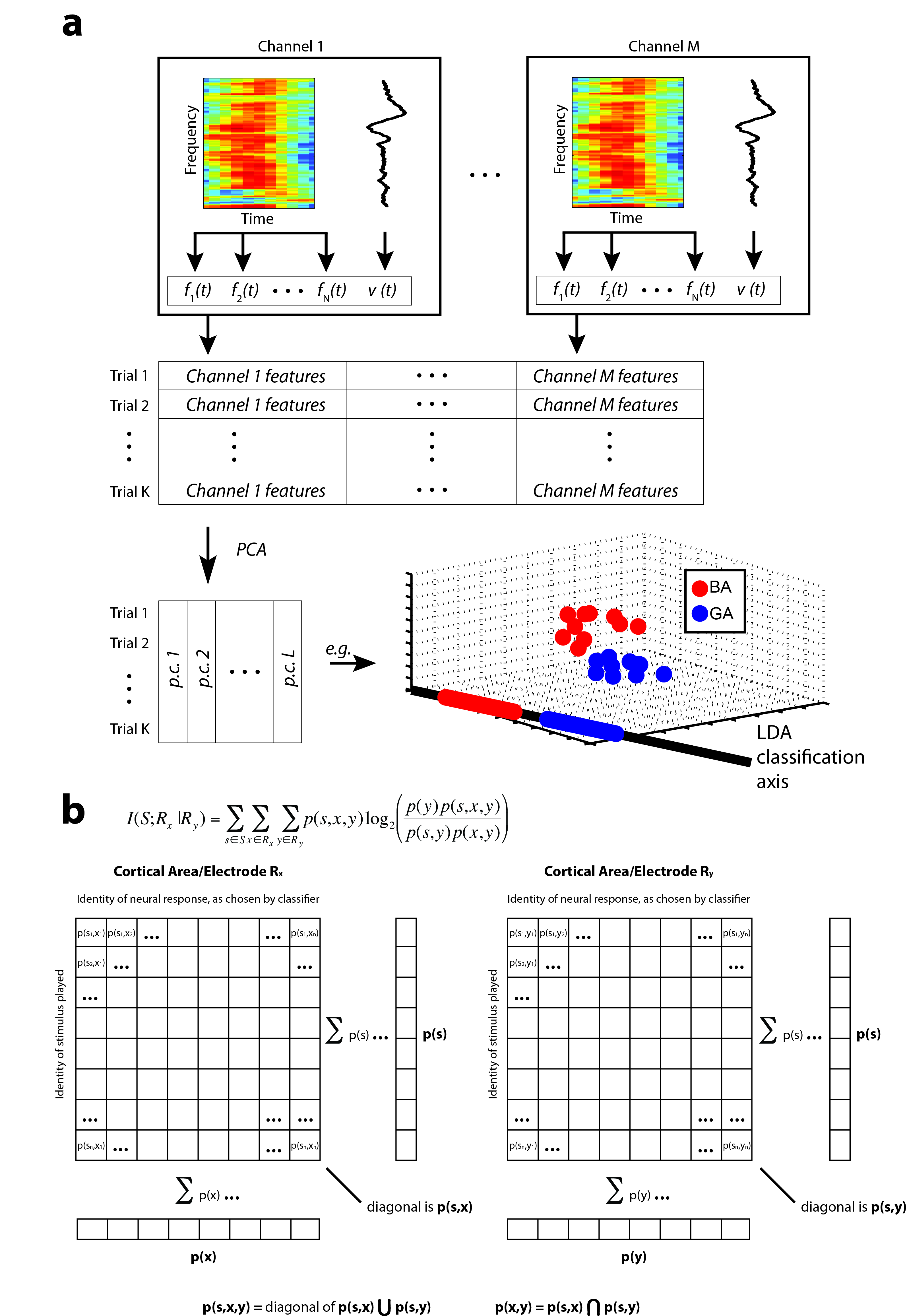

Supplement: Figure S1 — a, A visual description of the statistical classifier used to classify stimulus identity. We began with spectrograms and voltage traces for each trial over all channels. All neural features were unwrapped along the trials dimension, and principal components analysis (PCA) was applied to this matrix. The PCA reconstruction determined from the training set was then applied to the testing set and linear discriminate analysis (LDA) was used to classify the identity of the syllable for each trial. b, Derivation of probability distributions for conditional mutual information analyses, taken from pairwise classification frequencies. A confusion matrix of pairwise classification frequencies was generated for each electrode and divided by the total number of trials to generate probability distributions for the conditional mutual information equation as shown above. (TIF) [file pone.0073148.s001.tif]
